# Comparison of a User-Centered Design, Self-Management App to Existing mHealth Apps for Persons Living With HIV

**DOI:** 10.2196/mhealth.4882

**Published:** 2015-09-18

**Authors:** Rebecca Schnall, Jocelyn Patterson Mosley, Sarah J Iribarren, Suzanne Bakken, Alex Carballo-Diéguez, William Brown III

**Affiliations:** ^1^ Columbia University School of Nursing New York, NY United States; ^2^ National Center for HIV/AIDS, Viral Hepatitis, STD, and TB Prevention Centers for Disease Control and Prevention Atlanta, GA United States; ^3^ Columbia University Department of Biomedical Informatics New York, NY United States; ^4^ HIV Center, Division of Gender, Sexuality and Health New York State Psychiatric Institute and Columbia University New York, NY United States

**Keywords:** mHealth, HIV, mobile apps, user-centered design

## Abstract

**Background:**

There is preliminary evidence that mobile health (mHealth) apps are feasible, attractive, and an effective platform for the creation of self-management tools for persons living with HIV (PLWH). As a foundation for the current study, we conducted formative research using focus groups, participatory design sessions, and usability evaluation methods to inform the development of a health management app for PLWH. The formative research resulted in identification of the following functional requirements of a mHealth app for self-management: (1) communication between providers and peers, (2) medication reminders, (3) medication log, (4) lab reports, (5) pharmacy information, (6) nutrition and fitness, (7) resources (eg, social services, substance use, video testimonials), (8) settings, and (9) search function.

**Objective:**

The purpose of this study was to conduct an ecological review of the existing apps for PLWH and to compare the functionality of existing apps with the app specifications identified in our formative work.

**Methods:**

We searched two mobile app stores (Google Play and iTunes) and found a total of 5606 apps. We reviewed the apps, narrowed our search terms, and found a total of 112 apps. Of these, we excluded 97 (86.6%) apps that were either not in English (10/112, 8.9%), not HIV focused (32/112, 28.9%), or focused only on HIV prevention (2/112, 7.8%); targeted health care providers (26/112, 23.2%); provided information only on conference schedules and events (7/112, 6.3%), fundraisers (7/112, 6.3%), specific clinics (7/112, 6.3%), international or narrow local resources (3/112, 2.7%); or were identified in the first search but were no longer on the market at the next review (4/112, 3.6%). The 15 apps meeting inclusion criteria were then evaluated for inclusion of the nine functionalities identified in our earlier work.

**Results:**

Of the 15 apps that we included in our final review, none had all of the functionalities that were identified in our formative work. The apps that we identified included the following functionalities: communication with providers and/or peers (4/15, 27%), medication reminders (6/15, 40%), medication logs (7/15, 47%), lab reports (5/15, 33%), pharmacy information (4/15, 27%), resources (7/15, 47%), settings (11/15, 73%), and search function (6/15, 40%). No apps included nutrition or fitness information.

**Conclusions:**

Currently, there are only a small number of apps that have been designed for PLWH to manage their health. Of the apps that are currently available, none have all of the desired functionalities identified by PLWH and experts in our formative research. Findings from this work elucidate the need to develop and evaluate mobile apps that meet PLWH’s desired functional specifications.

## Introduction

Self-management is a dynamic, interactive, and regular process in which individuals manage their illness to promote health [1]. Through self-management an individual manages symptoms, treatments, lifestyle changes, and the consequences of health conditions [2] to maintain an adequate quality of life [3]. Self-management is increasingly important in chronic conditions, as the impact of these diseases continues to increase and affect over half of the US population [4]. Interventions that enable self-management and provide patients with information and skills that enhance their ability to participate in their health care are increasingly recognized as an essential component of the management of chronic conditions.

Chronic disease self-management is particularly relevant for persons living with HIV (PLWH) in the United States given that, as a result of advances in treatment regimens, HIV is now largely recognized as a chronic disease [5]. HIV requires lifelong therapy and is often characterized by multiple comorbidities that can present unique problems for the delivery of health care. A networked and well-resourced health care system is required to identify people with HIV, link them to care, provide services, and address comorbidities and other complications [6]. Improving PLWH’s ability to self-manage their illness is key to achieving the aims of the National HIV/AIDS Strategy for the United States by increasing access to care, improving health outcomes for PLWH, and reducing HIV-related health disparities [7].

HIV has disproportionately affected persons from underserved communities, specifically racial and ethnic minorities and those from low-socioeconomic groups [8-10]. Findings from the National HIV Behavioral Surveillance survey indicated that HIV prevalence was 2.8% among blacks and 1.2% among Latinos. HIV prevalence was higher among those who reported less than a high school education, compared with those with a high school education. HIV prevalence was also higher for those with an annual household income less than US $10,000, compared with those with an income of US $20,000. Geographic differences were also noted with prevalence being highest among survey participants in the Northeast followed by the southern region of the United States [11].

The potential for information and communication technology, such as mobile health (mHealth) technology and specifically mobile apps, to enhance self-management through the provision of support (information, education, reminders, etc) for behavior change has been well documented over the last decade [12-19]. A growing body of research confirms the benefits of empowering consumers with information and decision-making support [20-22]. Patient participation in their health has been shown to lead to increased patient satisfaction and positive changes in adherence patterns that translates into improved clinical outcomes [23-25].

Health disparities can be narrowed through the use of mHealth technologies, which are being used for patient monitoring, data collection, health information exchange, and real-time care management [26,27]. mHealth may be particularly relevant to low-socioeconomic PLWH since it has the potential to improve access to care by reducing challenges associated with geographic and economic disparities [28,29]. mHealth also provides unique possibilities for bridging the divide in health care delivery among underserved racial and ethnic minority groups [30]. The fact that African Americans and Hispanics download apps more frequently than non-Hispanic whites is a notable illustration [31].

Although mHealth tools for PLWH have been suggested as having potential to positively impact self-management, to date they have not been well developed or evaluated. Currently, there are only a small number of mHealth apps for PLWH [32], but little documentation exists showing that they have been developed in partnership with the target end users or health care providers to ensure that desired content and features are included in the app. Of the limited number of studies specifically focused on mobile apps for PLWH, only one study incorporated users’ preferences into the mobile app among HIV-positive young mothers [33], *but did not* rigorously evaluate the app after its development. In another study, researchers developed a mobile app consisting of a music program to improve adherence to antiretroviral (ARV) medications for adult PLWH, but did not report engaging the intended end users in its development [34].

Mobile phone apps are increasingly being used for the care of persons living with HIV and other sexually transmitted diseases (STDs), however most have failed to attract user attention and positive reviews. For example, in a review of existing apps for the care of persons living with HIV and other STDs and the prevention of these diseases, researchers found that apps were infrequently downloaded (ie, median 100 to 500 downloads) and not highly rated (ie, an average customer rating of 3.7 out of 5 stars). Based on this 2013 review, less than 0.3% of the more than 29,000 health-related apps available for iPhone and Android consumers were dedicated to HIV/STD information and prevention [32].

In response to the dearth of HIV apps derived from end users’ needs and design preferences, we conducted a multistage study to inform the design of a mobile app for PLWH to self-manage their illness. We then conducted an ecological review of existing apps with the same purpose—to support self-management for PLWH—to compare included functionalities. The goal of this paper is to report the design specifications of a mobile app that were derived from user-centered design methods and to compare the mobile app that we designed to the existing mobile apps that are currently available for PLWH to self-manage their disease.

## Methods

### App Design Process

#### Overview

The information systems research (ISR) framework [35] informed a three-stage process used to develop a design document outlining the blueprint of a mHealth self-management app for PLWH in the United States. The purpose of this project was to identify the desired features, user interface, and functionality of a mobile app for PLWH.

Focus groups, end-user design sessions, heuristic evaluation, and end-user usability testing were the methods applied to develop the design document. First, we conducted six focus group sessions with PLWH (n=50), ages 18 to 59 years, and three focus group sessions with HIV care providers (n=30) to identify the desired content, features, and function of the ideal mHealth app for PLWH to self-manage their health. Four group sessions with PLWH were conducted in English, and two group sessions with PLWH were conducted in Spanish. Among the six focus groups conducted with PLWH, over half of the participants reported their race as black/African American (26/50, 52%) and half reported their ethnicity as Latino/Hispanic (25/50, 50%). The methods for focus group data collection are published elsewhere [36].

Next, we conducted two participatory design sessions. In the first group, we had 5 participants out of 50 (10%); 3 of the 5 (60%) reported their race as African American and 2 (40%) reported their ethnicity as Latino. In the second design session, we had 6 participants out of 50 (12%), 3 (50%) of whom had participated in the first design session. Of the 6 participants, 3 (50%) were female and 3 (50%) were male; 4 (67%) participants self-identified as African American and 2 (33%) identified as Latino. Details of the methods and findings from the design sessions are published elsewhere [37].

Following these sessions, we created an initial low-fidelity prototype with a visual framework of the screen content and layout of the app in PowerPoint. To identify features that could increase technology acceptance, we conducted two types of usability assessments [38]: (1) a heuristic evaluation of the prototype using informaticians with experience in interface design and/or human computer interaction and (2) end-user usability testing systematically observing how well PLWH used the app. We had a total of five heuristic evaluators and 10 end-user usability testers. We used an iterative process revising the mock-up; after one heuristic evaluator and two subsequent end users evaluated the mock-up, we made changes to the app design in accordance with the recommendations. In total, we had five versions of our mock-ups. At the end of our usability testing, we finalized the design document which included the functional specifications and user interface design of a mHealth app for PLWH to self-manage their illness. Our end-users across groups and stages of the project had many similar ideas; there was a strong degree of agreement among all participants. We reached saturation of ideas before moving on to the next stage of app development.

Based on our user-centered iterative design process, our end users identified nine functionalities as being components of their ideal mobile app to support their health management needs. The functionalities included the following: communication, reminders, medication logs, lab reports, pharmacy information, nutrition and fitness, resources, settings, and search. The functionalities are listed in Table 1 and described in detail below.

**Table 1:** User-centered design self-management app for persons living with HIV: functionalities and sample details.

Functionality	Sample details
Communication	Provider-patient communication and peer communication
Reminders	Medication reminders for a time and date, setting medical appointment reminders, medical checklist (eg, flu vaccines, colonoscopy), and an audio recorder
Medication logs	Pill identification and summary of current and discontinued medications
Lab reports	CD4^a^ count, viral load, STD^b^, glucose, and CBC^c^ results and trends
Pharmacy information	Current pharmacy information
Nutrition and fitness	Nutrition and exercise information, food, and weight-loss tracking
Resources	HIV medical care, social services, substance use, law/advocacy, and case management
Settings	Profile picture, password, and alerts
Search	Search within the app

^a^Cluster of differentiation 4 (CD4): glycoprotein found on surface of T helper immune cells.

^b^Sexually transmitted disease (STD).

^b^Complete blood count (CBC).

#### Communication

Communication included both provider-patient communication and peer communication. Communication with medical providers included provider discussion forums, a 24/7 hotline, and a chat with a provider or case manager. Communication with peers included peer forums and interactive blogs.

#### Reminders

There were a number of functionalities associated with setting reminders, including medication reminders for a time and date, setting medical appointment reminders, medical checklist (eg, flu vaccines, colonoscopy), and an audio recorder.

#### Medication Logs

In our final design document, there is also a medication log which includes pill identification with the names and pictures of medications, a summary of current medications, medications discontinued, and specifically highlighting which medications had been discontinued in the past 3 months.

#### Lab Reports

Lab reports included CD4 count/viral load with a graph of the individual’s trends, sexually transmitted disease results, glucose trends, and complete blood count results.

#### Pharmacy Information

Participants wanted to record their current pharmacy location and contact information.

#### Nutrition and Fitness

Formative research participants wanted nutrition information, weight-loss goals, and a food tracker with a food diary and calorie calculator. Participants wanted physical activity information related to aerobic and muscle-strengthening activities.

#### Resources

Participants identified a number of broad categories of resources including information on HIV medical care, social services, substance abuse, and law/advocacy, as well as a sexual risk calculator, instructions on how to use a condom, HIV video testimonials, and an HIV medical dictionary. HIV medical care resources included HIV medical providers and clinics, HIV organizations/adult day programs, case management, HIV/AIDS Services Administration (HASA), AIDS medication assistance, mental health providers, support groups, and Medicaid information. Social services included food pantries, realtors for HASA recipients, and homeless services. Participants identified types of substance abuse resources and organizations such as needle exchange programs and support groups. Participants also wanted specific legal information about HIV transmission for each state and legal information about domestic violence. They also wanted video testimonials specific to women, men, and lesbian, gay, bisexual, and transgender (LGBT) persons, as well as testimonials about disclosing one’s status.

#### Settings

Participants wanted the app to have settings which allowed them to post a profile picture, set a password, and provide news, information, and medical alerts (eg, missed medications, appointments).

#### Search

Participants also specified that the app should have a search function to easily navigate through the app.

### Review of Existing Apps and Comparison to our User-Centered Design App

#### App Search Strategy

To identify health management apps for PLWH available in the marketplace we searched two online mobile app stores in March 2015—Google Play and iTunes—using the following search terms: HIV, AIDS, medication tracker, medication reminder, medication alarm, medication information, antiretroviral therapy (ART), antiretroviral adherence, ARV, adherence, AIDS treatment, HIV test, and pill.

After initially using these broad search terms, we did a review of the apps and found that many were not relevant and that a large number of apps were games, non-English, and had to do with first aid or the provision of aid. As a result, we narrowed our search terms to the following: HIV treatment, HIV medication, HIV/AIDS treatment, HIV/AIDS care, HIV reminder, AIDS medication, antiretroviral, and living with HIV. Each term was searched separately in each store and a list of search results was compiled.

#### Selection Criteria

The apps were eligible for inclusion if they were targeted to PLWH. The apps were excluded if they were games, not in English, for health care professionals, for fundraising, not focused on HIV, a specific clinic, international (eg, Hong Kong medications), local resources (eg, only for the city of Philadelphia), or a conference itinerary. Two study team members (RS, JPM) independently reviewed the titles of each of the apps and excluded apps from further review that clearly did not meet eligibility criteria.

#### Data Extraction, App Selection, and Assessment

**Figure 1:**
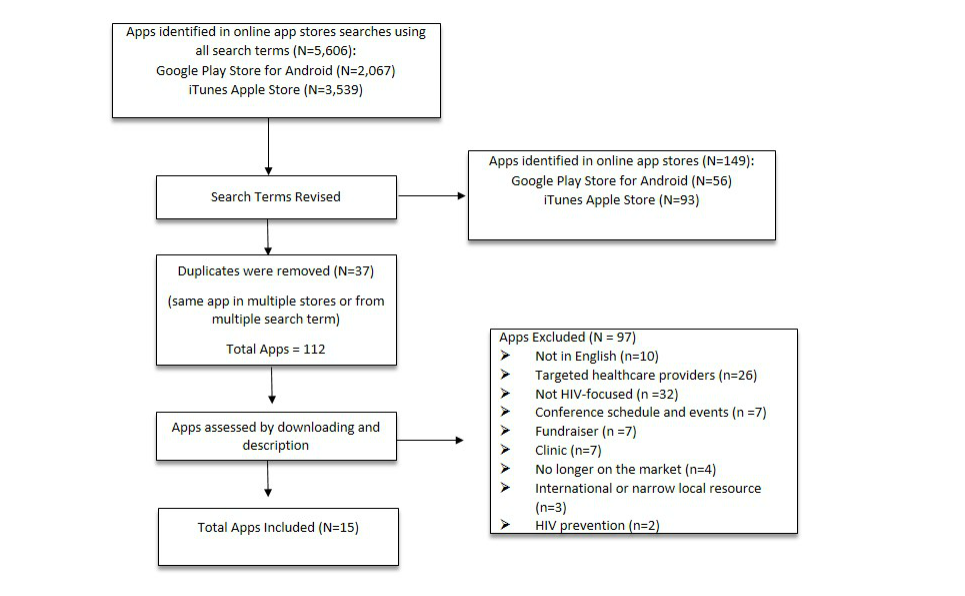
Flowchart of the screening process of mobile apps for persons living with HIV to manage their health.

We identified a total 149 apps from both app stores using the terms listed above. We then assessed the list of apps to identify any duplicate apps (ie, same app available in Android and Apple platforms). We found 37 apps that were the same in both stores, narrowing our total number of apps to 112 (see Figure 1). Of these, we excluded 97 (86.6%) apps that either were not in English (10/112, 8.9%), not HIV focused (32/28.6%), or focused only on HIV prevention (2/112, 1.8%); targeted health care providers (26/112, 23.2%); provided information only on conference schedules and events (7/112, 6.3%), fundraisers (7/112, 6.3%), specific clinics (7/112, 6.3%), or international or narrow local resources (3/112, 2.7%); or were identified in the first search but were no longer on the market at the next review (4/112, 3.6%).  At the end of our extraction process, we identified a total of 15 unique apps.

The apps meeting eligibility criteria were downloaded for full functionality evaluation by two of the study authors (JPM, SJI). A standardized form was created to extract app characteristics using Research Electronic Data Capture (REDCap). The REDCap Web-based application allows users to build and manage online surveys and databases quickly and securely [39]. Each app was assessed for platform (ie, Apple or Android), targeted population (ie, men who have sex with men [MSM], men, women, youth/adolescents, young adults, older adults, and homeless persons), user rating and number of people contributing to the rating, range of the number of downloads where available, and the cost to download (see Multimedia Appendix 1). Finally, we coded each app to determine if it had the functionality identified in our formative work. Broader functionalities included the following: communication (ie, peers and medical provider), setting medication reminders, medication logs, lab reports, pharmacy information, nutrition and fitness, resources, and settings.

## Results

Of the 15 apps included in our comparative assessment, 9 (60%) were available on both platforms, 5 (33%) were only available from iTunes, and 1 (7%) was only available from Google Play. All of the apps were available for free to download (see Table 2).

Of the 8 out of 15 (53%) apps with customer ratings, the weighted average rating was 4.42 (SD 2.10) stars. The mean number of reviews for rated apps was 36 (SD 41) (range 1-91). Only Google Play provides information on the number of downloads per app. Of the 10 apps with download information, 2 (20%) apps had 100 to 500 downloads, 2 (20%) apps had 500 to 1000, 2 (20%) apps had 1000 to 5000 downloads, and 2 (20%) apps had 5000 to 10,000 downloads. None of the apps were focused on any of the targeted populations listed above.

Most (10/15, 67%) of the apps were available in English only. Out of the 15 apps, 1 (7%) was available in English and Spanish, and 2 (13%) were available in English and French. Of the 15 apps, 1 (7%) was available in English, German, and Spanish and 1 (7%) was available in four languages: English, French, German, and Spanish.

None of the apps included all of the functions that were identified in our formative work as specified by end users. The following functionalities were included in the apps in our review: communication with providers and/or peers (4/15, 27%), medication reminders (6/15, 40%), medication log (7/15, 47%), lab reports (5/15, 33%), pharmacy Information (4/15, 27%), resources (7/15, 47%), settings (11/15, 73%), and search function (6/15, 40%). No apps included functionality related to nutrition or fitness. Out of 15 apps, 5 (33%) included four or more functions, while the remainder had less than four.

**Table 2:** Overview of 15 apps for persons living with HIV.

App name	Author	Platform	Rating (# rating)	Down-loads, n	Languages supported	User-centered design HIV app functionality
AIDSinfo HIV/AIDS Drug Database [40]	NLM^a^ at NIH^b^	Google, Apple	4.2 [14]	500-1000	English	Settings, medication reminders, search
AIDSinfo HIV/AIDS Glossary [41]	NLM at NIH	Google, Apple	4.2 [42]	500-1000	English, Spanish	Resources (HIV medical dictionary), search
aidsmap news [43]	Thomas Paterson	Apple	N/A^c^	N/A	English	Settings (alerts: HIV news), search
Facing AIDS [44]	HHS^d^	Google, Apple	5 (Google-1^e^)	100-500	English	Communication (peers), settings (profile picture)
HIV Answers [45]	Gilead Sciences	Google, Apple	4.4 (Google-18)	1000-5000	English	Medication log, pharmacy information, resources (HIV medical care, social services), settings (password), search
HIV Medication Guide [46]	Headcan	Apple	N/A	N/A	English, French	Search
HIV Testing Sites & Care Services Locator [47]	HHS	Apple, Google	3.7 (Google-3)	100-500	English	Resources (HIV medical care, social services, substance use), search
HIVPlus Treatment Guide [48]	Here Media	Apple, Google	3.5 (Apple-8) 4.6 (Google-29)	1000-5000	English	Medication reminders (date, recorder, medical appointments), medication log, lab reports, resources (HIV video testimonials, medical dictionary), settings (HIV info, HIV news, missed appointment alerts)
iStayHealthy [49]	Peter Schmidt	Apple, Google	4.6 (Google-91)	5000-10,000	English, French, German, Spanish	Medication reminders (date), medication log (pill ID, summary), lab reports (glucose, CBC^f^, CD4^g^ count/viral load) pharmacy information, resources (HIV medical care), settings (alerts)
My Health Matters [50]	Merck	Apple	N/A	N/A	English, German, Spanish	Medication reminders (date, recorder), medication log, lab reports (glucose, CBC, CD4 count/viral load), pharmacy information, resources, settings (password)
NV SelfCare [51]	The Center Las Vegas	Apple, Google	N/A	10-50	English	Communication (providers), medication log, settings (username, password)
Red Ribbon, Your HIV/AIDS Health Manager [52]	Communi-cation Software, Inc	Android	3.7 (Google-6)	100-500	English	Medication reminders (date), medication log, lab reports, settings (username, password)
TalkPositive [53]	Pharmi- Web2002 Limited	Apple	N/A	N/A	English	Medication reminders (date, medical appointments), medication log, lab reports, pharmacy information, settings (username)
The Body [54]	Remedy Health Media	Apple, Google	4.4 (Google-87) 5 (Apple-9)	5000-10,000	English	Communication (peers), resources (HIV video testimonials), HIV news
YourDocTalk: HIV Treatment Talking Tool [55]	CATIE^h^	Apple	N/A	N/A	English, French	Communication (providers)

^a^National Library of Medicine (NLM).

^b^National Institutes of Health (NIH).

^c^Not applicable (N/A).

^d^US Department of Health and Human Services (HHS).

^e^This number denotes the number of people who provided reviews.

^f^Complete blood count (CBC).

^g^Cluster of differentiation 4 (CD4).

^h^Canadian AIDS Treatment Information Exchange (CATIE).

## Discussion

### Principal Findings

Wide gaps exist between user-desired functionality of apps and those which currently exist for PLWH. As described earlier, many of the desired functionalities, such as lab reports, communication tools, and nutrition and fitness components, did not exist in many of the existing apps for PLWH. It has been well documented in the technology acceptance literature that if technology is not perceived as useful then it is unlikely that end users will use it [56,57]. This points to the need to develop technology, and more specifically apps, which meet the desired functional specifications of the intended end users. Given these current gaps, it is not surprising that many apps leave the marketplace quickly and are not widely downloaded (see Table 2).

In addition to the gaps in functionality, most apps are only available in English. There is a dearth of apps available in Spanish (3/15, 20%). This is particularly relevant since Hispanics/Latinos accounted for over 20% of all new HIV infections in the United States despite representing only 16% of the total US population [8]. This points to the need for the development of existing apps in Spanish and is an especially important charge for the apps developed by the US government.

### Rapidly Changing Landscape for mHealth Apps

By some estimates, there are as many as 97,000 health apps currently available in the app stores [58]. Of these, there are a limited number of apps available that are specifically tailored for PLWH to manage their health. The landscape for mHealth apps is also rapidly changing. For example, in a 2013 review of apps for HIV/STD-positive persons, Muessig et al identified 8 apps for PLWH. Of those apps identified in their review, only 5 are still available [32]. Similarly, in our review 4 of our originally identified apps were no longer available when the second reviewer went to download the app a few weeks later. The rapid turnover of available apps points to issues with regulation, sustainability, and maintenance. There is currently much debate as to which apps need to be regulated and by whom [59]. Of more relevance to our work, there is greater concern over sustainability since it is clear that apps rapidly leave the marketplace, which can be very problematic if patients successfully rely on an mHealth app to support the management of their illness. Lack of sustainability also presents challenges for rigorous evaluation of existing apps because of their rapid disappearance.

### US Food and Drug Administration Regulation of Apps

The US Food and Drug Administration (FDA) has a growing interest in the regulation of certain types of medical apps in the United States. The most recent FDA guidance refers only to mobile medical apps. More specifically, the FDA will apply its regulatory oversight to mobile apps that are medical devices and whose functionality could pose a risk to a patient’s safety if the mobile app were to function as intended [60]. Current regulation of apps is important to consider in thinking about dissemination, oversight, cost to development, and use of mobile apps. Given the current guidelines, the app that we designed in our formative work would not be subject to FDA oversight. Moreover, the apps that we identified in our review also do not fall within the category of mobile medical apps.

### Apps for Persons Living With HIV

While there is a dearth of apps available specifically for PLWH, there are a number of apps that are available for persons to self-manage their health that are not unique to HIV. For example, in a review of mobile apps for supporting medication self-management, 424 apps were identified, pointing to the broad availability of these apps, but findings from this review pointed to the same limitations as those found in our study: the quality, content, and functionality are highly variable [42,61]. Importantly, medication self-management apps may not need to be specifically designed for PLWH and, consequently, some of those 424 apps may be relevant to our study population. Even so, the review found that most apps were unable to support complex or varying regimens which may make them useless to PLWH who need to change their regimens in response to resistance, and who are also frequently being treated for opportunistic infections and/or comorbid conditions [62,63].

Findings from our formative work supported the design and creation of an app for PLWH which would provide an entire set of desirable functions. Our formative work focused on the creation of a “one-size-fits-all" app for PLWH. There are some users who may prefer simpler apps that only provide specific functions limited to what they want. In addition, large multifunction apps take up a lot of storage space on devices. Nonetheless, our formative work did not ask users to consider space limitations and instead asked them to focus on the components and user interface which would make the app most useful and easiest to use.

In our formative work, our study participants identified a number of functionalities beyond medication management that they considered key to the management of their disease. Findings from our work are further supported by the current demographics of the HIV epidemic in the United States. HIV in the United States has become largely a chronic illness [63]. PLWH are living longer, and as they live longer they are experiencing chronic diseases similar to their HIV-negative counterparts. They have even greater needs for self-managing their health [64], illustrating the need for the development of a self-management app for PLWH that we identified in our formative work.

In terms of functionality, more than half of the apps in our review included settings and nearly half included medication logs, reminders, resources, and a search function. Yet other features such as communication with providers and/or peers, pharmacy information, and lab reports were less available. This is unfortunate since the functionalities were described as desired by our targeted end users. Interestingly, PLWH and health care providers for PLWH identified nutrition and fitness as key content necessary for maintenance of their health. Diet and fitness have been identified by end users as important health information needs in previous studies [65]. At the same time, none of the HIV apps that we identified in our review included this content.

### Limitations

There are some important limitations to mention. First, the formative research phase that we conducted used relatively small samples in a single geographic area. Therefore, the views are not necessarily representative of all English- and Spanish-speaking PLWH. This is an important limitation to our formative research results and therefore also an important limitation to the existing app-feature assessment results presented in this paper. Nonetheless, we did include both English and Spanish speakers in our design process, as well as health care providers, to gather more generalizable information.

There are also limitations with our review of the existing apps. First, the search functions within the mobile app store are limited and a search term can return hundreds or thousands of unrelated apps. Second, other apps were identified as relevant and meeting inclusion criteria. However, at the stage of review some were found to be no longer available. Therefore, their features could not be assessed for inclusion in this study. Because user reviews are not included for all apps we were only able to provide user review scores on a portion of the included apps.

### Conclusions

Our formative work used an iterative design process incorporating end-user feedback and expert review to identify the functional specifications and user interface design of a design document for an mHealth app for PLWH to self-manage their health. Development of mHealth technologies is currently progressing at a rapid pace that is evident by the large number of apps that are currently available. Given the low uptake and rapid disappearance of apps, there is a strong need to integrate the needs of the end users in technology development. Findings from our review demonstrate the gap between what PLWH identified as the functional components of an app they desired versus the functionalities within apps that currently exist. Future work is needed to develop apps that meet end users' desired needs. Furthermore, there is a need for rigorous evaluation of these apps to determine their efficacy to improve health outcomes. Finally, if an app were found to be useful and usable from the end users' perspective, as well as effective at improving health outcomes, then a model for sustainability needs to be developed so that these apps can be appropriately disseminated and implemented in the lives of persons who suffer from chronic illnesses such as HIV.

## Appendix

Multimedia Appendix 1 Standardized form to extract app characteristics.

## Abbreviations

ART: antiretroviral therapy
ARV: antiretroviral
CATIE: Canadian AIDS Treatment Information Exchange
CBC: complete blood count
CD4: cluster of differentiation 4
FDA: US Food and Drug Administration
HASA: HIV/AIDS Services Administration
HHS: US Department of Health and Human Services
ISR: information systems research
LGBT: lesbian, gay, bisexual, and transgender
MSM: men who have sex with men
N/A: not applicable
NIH: National Institutes of Health
NLM: National Library of Medicine
PLWH: persons living with HIV
REDCap: Research Electronic Data Capture
STD: sexually transmitted disease
