# Supplementary material for: Comparison of a User-Centered Design, Self-Management App to Existing mHealth Apps for Persons Living With HIV
Source: JMIR Mhealth Uhealth. 2015 Sep 18;3(3):e91. doi: 10.2196/mhealth.4882 (PMC4704937; doi:10.2196/mhealth.4882)
Supplement: Multimedia Appendix 1 [file mhealth_v3i3e91_app1.pdf]

Multimedia Appendix 1. Standardized Form to Extract App Characteristics

Record ID			
App Name			
Search Term	HIV/AIDS treatment<br>HIV/AIDS care<br>HIV treatment	HIV medication<br>AIDS medication<br>HIV reminder	antiretroviral<br>living with HIV
Price			
Rating			
Downloads			
Targeted Population	MSM<br>Men<br>Youth/ Adolescents	Young Adults<br>Older Adults<br>Women	Homeless<br>None
Platform	Apple	Android	
Communication	o Yes	o No	
Communication Options			
Setting My Medication Reminders	o Yes<br>o No		
Setting My Medication Reminders Options	Set Date of Medication Reminder<br>Setting My Medical Appointment Reminders	My Medical Checklist Due Today<br>Recorder	
My Medication Log	o Yes	o No	
My Medication	Pill ID		

Log Options	Summary of My Medication Log	
My Lab Reports	o Yes<br>o No	
Lab Reports Options	Glucose/ CBC<br>STDs	CD4/ viral load
My Pharmacy Info	o Yes	o No
Nutrition and Fitness	o Yes	o No
Nutrition and Fitness Options	Nutrition<br>Physical Activity	
Resources	o Yes	o No
Resources – Options	HIV medical Care<br>Social Services<br>Substance Use<br>Law/Advocacy	My Sexual Risk Calculator<br>How to Use a Condom<br>HIV Video Testimonials<br>HIV medical dictionary
Settings	o Yes	o No
Settings - Options	User Name<br>Password Profile picture Alerts: HIV Info<br>Alerts: HIV news	Medical Alerts: Medications<br>Missed Medical Alerts: medication refills<br>Medical Alerts: missed appointments
